# Risk factors of mobile phone use while driving in Queensland: Prevalence, attitudes, crash risk perception, and task-management strategies

**DOI:** 10.1371/journal.pone.0183361

**Published:** 2017-09-06

**Authors:** Oscar Oviedo-Trespalacios, Mark King, Md. Mazharul Haque, Simon Washington

**Affiliations:** 1 Australia Queensland University of Technology (QUT), Centre for Accident Research and Road Safety – Queensland (CARRS-Q), Queensland, Australia; 2 Australia Queensland University of Technology (QUT), Institute of Health and Biomedical Innovation (IHBI), Faculty of Health, Queensland, Australia; 3 Australia Queensland University of Technology (QUT), Civil Engineering and Built Environment, Science and Engineering Faculty, Queensland, Australia; 4 School of Civil Engineering, The University of Queensland, Queensland, Australia; University of Rijeka, CROATIA

## Abstract

Distracted driving is one of the most significant human factor issues in transport safety. Mobile phone interactions while driving may involve a multitude of cognitive and physical resources that result in inferior driving performance and reduced safety margins. The current study investigates characteristics of usage, risk factors, compensatory strategies in use and characteristics of high-frequency offenders of mobile phone use while driving. A series of questions were administered to drivers in Queensland (Australia) using an on-line questionnaire. A total of 484 drivers (34.9% males and 49.8% aged 17–25) participated anonymously. At least one of every two motorists surveyed reported engaging in distracted driving. Drivers were unable to acknowledge the increased crash risk associated with answering and locating a ringing phone in contrast to other tasks such as texting/browsing. Attitudes towards mobile phone usage were more favourable for talking than texting or browsing. Lowering the driving speed and increasing the distance from the vehicle in front were the most popular task-management strategies for talking and texting/browsing while driving. On the other hand, keeping the mobile phone low (e.g. in the driver’s lap or on the passenger seat) was the favourite strategy used by drivers to avoid police fines for both talking and texting/browsing. Logistic regression models were fitted to understand differences in risk factors for engaging in mobile phone conversations and browsing/texting while driving. For both tasks, exposure to driving, driving experience, driving history (offences and crashes), and attitudes were significant predictors. Future mobile phone prevention efforts would benefit from development of safe attitudes and increasing risk literacy. Enforcement of mobile phone distraction should be re-engineered, as the use of task-management strategies to evade police enforcement seems to dilute its effect on the prevention of this behaviour. Some countermeasures and suggestions were proposed in the design of public education campaigns and driver-mobile phone interaction.

## Introduction

It is widely recognised that road trauma remains a burden for global health. In 2015, the World Health Organization estimated that 1.2 million people die as a result of road crashes worldwide annually [1]. The premise that human factors are the root cause of most road safety safe-critical events and therefore should be the target of safety and prevention efforts is widely recognised. Mobile phone interactions while driving involve a multitude of cognitive and physical resources, with such behaviour consistently linked with inferior driving performance and reduced safety margins [2, 3]. Conservative estimates have shown that the crash risk is higher among distracted drivers than non-distracted drivers. In the USA alone, mobile phone distraction is likely to explain a quarter of crashes [4]. This is confirmed in the largest naturalistic study worldwide, Second Strategic Highway Research Program Naturalistic Driving Study (SHRP 2 NDS), which has reported data showing that hand-based mobile phone interactions increase crash risk, with odds ratios indicating that texting increases crash risk by ∼6.1 [5].

At first glance, a combination of a definitive mobile phone ban while driving and strong enforcement should be the most effective approach to address this problem. However, research worldwide (e.g. Farmer, Klauer [6]) showing a large prevalence of mobile phone distracted driving has confirmed that legislation and enforcement is not necessarily preventing mobile phone usage while driving. Pioneers and advanced road safety systems with strong laws on distracted driving such as Australia and the USA have been unsuccessful in stopping these behaviours. Recent naturalistic driving estimates in the USA have confirmed that drivers are distracted in some way around half of the time, with mobile phone use making up about a quarter of the distracted time [5]. Observational on-road studies have found that nearly 18.7% in the USA [7] and 5% in Australia [8] use a mobile phone while driving.

Some groups of the population seem more susceptible to mobile phone distraction than others. Alarmingly, distracted driving, particularly the use of mobile phones while driving, is more prevalent among young drivers aged 18–24 years. In Australia, Young, Rudin-Brown (8) confirmed through an observational study that young drivers (<30 years) more frequently use handheld mobiles than middle-aged and older drivers (30 years and more) while driving. This is in accordance with international studies, where at least one in two young drivers in U.S. and Canada have been found to use a mobile phone while driving [9]. Consistently, multi-national studies have confirmed that mobile usage while driving, particularly handheld interactions, is associated with crash risk among young drivers [10]. There may be gender differences within young drivers: recently in Victoria (Australia), males were observed to have a larger engagement in mobile phone distracted activities than females [11]. A greater understanding of usage patterns and predictors among vulnerable groups will support the development of targeted countermeasures.

Mobile phone distracted driving is an increasingly prevalent and risky behaviour among drivers; however, few recent studies have investigated the prevalence in Queensland and the underlying beliefs held by drivers about engaging in conversations or texting/browsing tasks. Additionally, little information has been reported on usage patterns and potential task-management strategies adopted by drivers to avoid penalties or conflicts with the driving task. Thus, the current research aimed to:

1. Obtain self-reported behaviour information regarding the extent to which people use phones while driving, and explore patterns of mobile phone activity to determine whether there are differences in these and whether self-reported frequencies of use, risk perception and task-management strategies differ for different mobile phone tasks;
2. Explore personal, attitudinal, perceptual, and behavioural information that might provide insight into the factors influencing the target distracting behaviour (texting/browsing and handheld conversation) and thus inform the design of interventions to reduce mobile phone distracted driving.

## Method

The study protocol was approved by the Human Research Ethics Committee of the Queensland University of Technology (Approval Number: 1500001038).

### Recruitment

The survey was conducted as an anonymous online questionnaire. The survey was disseminated using social media (Twitter, Facebook, and blogs), local press releases, and electronic mail through Queensland University of Technology mailing lists and public face-to-face dissemination, with a brief advertisement providing an overview of the study (driving behaviours in general), a link to an on-line informed consent form and a link to the survey. The respondents were assured that participation was voluntary. Participants were informed that they would not be penalized if they chose not to participate or complete the survey. On average, a respondent took 25 minutes to complete the survey.

### Participants

A total of 484 drivers completed the survey. Of these respondents, 34.9% were males and 65.1% females. With respect to age group, 49.8% were aged 17–25 years and 50.2% were aged 26–65 years. The average time with a valid licence was 3.33 years (SD = 0.13) for the 17–25 years group, and 18.25 (SD = 0.72) for the 26–65 years group. In general, participants reported driving on average 1.33 hours (SD = 0.05) on a typical day. Some other important demographic characteristics of the sample are reported in Table 1. Most participants (71.9%) reported having driven 10 hours or least in the last week and use driving for a combination of work and personal purposes (57.4%). Small and medium cars are more frequently driven by the respondents (81.0%). A total of 120 (24.8%) respondents reported having at least one crash and 172 (35.5%) reported having at least one traffic offence in the last 3 years.

**Table 1:** Respondents’ demographic profile and driving patterns.

Measures	Frequency	Percentage
*How many hours driven per week (last 12 months)*		
Less than 5 hours per week	153	31.6%
6 to 10 hours per week	195	40.3%
11 to 20 hours per week	108	22.3%
21 to 30 hours per week	19	3.9%
More than 30 hours per week	9	1.9%
*Driving purpose*		
Mostly for work	69	14.3%
Mostly personal	137	28.3%
Mixture of work and personal	278	57.4%
*Type of vehicle (Usually drive)*		
SUV/Utility car	92	19.0%
Small/medium Car	392	81.0%
*Prior involvement in crashes (last 3 years)*		
Yes	120	24.8%
No	364	75.2%
*Prior involvement in traffic offences (last 3 years)*		
Yes	172	35.5%
No	312	64.5%

### Questionnaire measures

Besides sociodemographic characteristics and driving patterns described above, the survey contained five main sections:

#### Mobile phone use for talking while driving

Target behaviours were limited to sub-elements of handheld conversations while driving. Two items were used: “On a typical day have you spoken on a handheld phone while driving?” (yes-no scale) and “On a typical day have you located and answered a ringing phone while driving?” (yes-no scale). The frequency of the behaviours was measured by asking, if yes, “What percentage of your total drive time (0%-100%) did this take up?” for the handheld conversation and, “how many times did you engage in this behaviour per hour while driving?” for locating/answering a ringing phone. This research targets handheld conversations because these are illegal in Australia while hands-free devices are allowed (restrictions applied for novice drivers under 25 years). Additionally, the rationale for including a question on locating and answering a ringing phone is that this sub-component of a mobile phone conversation has been ranked among the riskiest mobile phone interactions with the odds of crash involvement being 4.8 times compared with 2.2 for handheld conversations [5]. Supplementary sub-components of handheld mobile phone conversations which entail highly intensive visual, cognitive, and manual interactions (e.g. dialling or battery/duration monitoring) are considered as browsing.

#### Mobile phone use for texting/browsing while driving

Firstly, one item was used to study engagement on texting and browsing: “On a typical day have you texted or browsed on your phone while driving?” These behaviours were combined because from a human factors perspective, these tasks involve similar demands for the driver. A distracted driver involved in direct use of a mobile phone performs two tasks: “transmission of information from the mobile phone to the driver” and “usage of mobile phone’s control mechanisms by the driver” [12]. Texting and browsing share the same transmission and usage interfaces (similar verbal codes, visual mode, cognitive processing, and manual responses), which make these tasks transposable in terms of driver workload (see Wickens [13]). Also, given that most of the research has agreed that visual demands are particularly risky for drivers [12], and definition of this usage typology might not be clear for drivers, a question about the driver taking their eyes off of the road was included: “On a typical day have you looked at a handheld phone while driving for more than 2 seconds continuously (yes-no scale)?” The frequency of the behaviours was measured by asking, if yes, “how many times in a one hour drive?” for both items. These behaviours were included due to a consistent link with higher crash risk with the odds of crash involvement being estimated as 2.7 times greater for browsing, 6.1 times for texting, and 12.2 times for mobile phone dialling [5]. In addition, crashes and near crashes have been found to increase monotonically after 2 seconds of taking the eyes off the road [14].

#### Attitudes towards mobile phone distracted driving

Mobile phone distracted driving safety attitudes were studied for two main behaviours, their typical engagement in mobile phone conversation and texting/browsing on handheld phone. No distinction was made between mobile phone conversation interfaces such as in-vehicle cell-phone, hands-free, handheld, or speaker; this was done for two reasons. First, we wanted drivers to answer based on their actual driving experience which contributed to and reflected the definition of their attitudes. Second, today there is a consensus in the literature that there is no major variation in crash risk between interfaces [15]. The questionnaire included eight items formulated for both talking or texting/browsing: (*i*) it is easy for someone to tell if their driving has been affected, (*ii*) I would need a lot of convincing to believe it is dangerous, (*iii*) the effects on driving ability are likely to be only very minor, (*iv*) the only people at risk are those who use a mobile while driving, (*v*) any distraction effects will last even after the task is finished, (*vi*) demanding driving conditions will prevent me from [*talking* or *texting/browsing*] on a mobile phone, (*vii*) presence of law enforcement and risk of a fine will prevent me from [*talking* or *texting/browsing*] on a mobile phone, and (*viii*) it is completely safe because I am generally extra careful. Responses were on a Likert scale ranging from 1 (“strongly disagree”) to 5 (“strongly agree”).

#### Risk perception towards mobile phone distracted driving

Perceived risk was measured in terms of perceived likelihood of crash risk. Questions were formulated independently for each one of the items used to measure engagement in mobile phone conversation and texting/browsing (see *Mobile phone usage for talking while driving and Mobile phone usage for texting/browsing while driving*): How likely are you to have a crash if you are using a mobile phone for …? (*i*) a voice call, (*ii*) texting/browsing, (*iii*) looking at the phone continuously for more than 2 seconds, and (*iv*) answering a ringing phone. Responses were on a Likert scale ranging from 1 (“very unlikely”) to 5 (“very likely”).

#### Task-management strategies in mobile phone distracted driving

Two main groups of task-management strategies were included in this study for mobile phone conversation and texting/browsing on a handheld phone (see *Attitudes towards mobile phone distracted driving* for the rationale). Firstly, participants were asked to report changes in driving behaviour due to mobile phone usage: “When you are using your mobile phone while driving, how likely is it that you would …?” (*i*) lower your driving speed, (*ii*) increase your distance from the vehicle in front, (*iii*) scan the environment more often, and (*iv*) increase the control over the steering wheel. These measurements are inclusive of behavioural adaptations reported in previous literature on mobile phone distracted driving (see Oviedo-Trespalacios, Haque (12)). Secondly, participants were asked to report behaviours performed to avoid police enforcement. “When you are using your mobile phone while driving, how likely is it that you would…?” (*i*) keep your mobile phone low (e.g. in lap or on passenger seat) for avoiding police, (*ii*) scan the environment for police, and (*iii*) cover the phone all the time with your hand. Although other ways and means of risk management are possible, e.g. pulling-over or using the phone while waiting at a signalised intersection, these strategies do not have a direct impact on road safety since the vehicle is not moving. Responses were on a Likert scale ranging from 1 (“very unlikely”) to 5 (“very likely”).

### Data and analysis methods

The self-reported driving behaviours, attitudes, risk perception, and task-management strategies response frequencies were calculated by mobile phone task. The data collected from subjects has been made available as a supporting file (S1 Dataset). Frequencies for mobile phone tasks were averaged and assessed as non-normal (variables were considered to be normal if absolute skewness was between -2 and +2 and absolute kurtosis was between -7 and +7). Therefore, to study relationships between frequencies of mobile phone tasks while driving, non-parametric tests were utilised when possible: Spearman’s *rho* and the Wilcoxon signed rank test. To study differences in perceived risk, attitudes, and self-reported use of task-management strategies between handheld conversations and texting/browsing, the McNemar—Bowker test for paired data was utilised. To examine the role of the respondents’ demographic profile, driving patterns, crash risk perceptions, attitudes, and self-reports of task-management strategies on mobile phone distracted driving on a typical day, two stepwise binary logistic regression models were tested using these outcomes as the dependent variable: “no” (0) and “yes” (1). We performed stratified analyses with separate models for handheld talking (any sub-component) and texting/browsing (any sub-component). To investigate potential bias in maximum likelihood estimation caused by low self-reported behaviours, models were re-fitted and compared using Firth's penalised likelihood procedure [16].

## Results

### Drivers’ general self-reported behaviour and attitudes towards mobile phone distracted driving

To address Aim 1, self-reported behaviour and attitudinal characteristics of mobile phone distracted drivers were analysed. Table 2 shows the prevalence of behaviour based on task subcomponents and usage frequency. Nearly 49% of drivers engaged in at least one of the sub-components of mobile phone conversations. Locating and answering a ringing phone was the most frequent task reported by almost 45% of the participants compared to the 28% of participants who reported speaking on a handheld phone. The average length of conversations among those who spoke over handheld phone was 9 minutes, and drivers who located and answered a ringing phone did this task on an average 1.5 times per hour of driving. The correlation between the frequencies of locating/answering a ringing phone and speaking on a handheld phone was statistically significant (Spearman’s rho, 0.20, *p <* 0.05, *n* = 117). Nearly 50% of drivers engaged in at least one of these sub-components of handheld mobile phone tasks related to conversations. Participants reported that, on a typical day, they texted or browsed with a lower frequency (34%) than they looked at the phone for more than 2 seconds (39%). Frequency of engagement was 3.5 times per hour driving for texting and browsing and 3.9 times per hour driving for looking at the phone for more than 2 seconds; however the differences between these two activities were not statistically significant (Wilcoxon signed rank test, 0.46, *p >* 0.05). The correlation between the frequencies of texting/browsing and looking at the phone for more than 2 seconds was statistically significant (Spearman’s rho, 0.66, *p <* 0.001, *n* = 109).

**Table 2:** Mobile phone use on a typical day and frequency.

Mobile phone tasks	Yes *n (%)*	No *n (%)*	*Exposure to the task*
*Mobile phone usage for talking (any sub-component)*	*235 (49)*	*249 (51)*	
Located and answered a ringing phone	219 (45)	265 (55)	1.51 (SD 1.6) times per hour of driving
Spoken on a handheld phone	137 (28)	347 (72)	9.18 (SD 11.7) minutes per hour driving
*Mobile phone usage for browsing/texting (any sub-component)*	*241 (50)*	*243 (50)*	
Texted or browsed on your phone	162 (34)	322 (67)	3.5 (SD 3.3) times per hour driving
Looked at a handheld phone while driving for more than 2 seconds	188 (39)	296 (61)	3.9 (SD 5.7) times per hour driving

Frequency of responses for self-reported risk perception is presented in Table 3. With regard the talking activity, most of the participants reported that crashes are likely while locating and answering a ringing phone (41%). However, most of the participants reported that crashes are very unlikely or unlikely while performing a handheld conversation (44%). On the other hand, most of the participants reported high perceived crash risk for mobile phone usage for browsing/texting (71–72%). Comparisons of the responses showed that all items have statistically significant differences in perceived risk crash.

**Table 3:** Perceived crash risk per mobile phone sub-task.

How likely are you to have a crash if you are using a Mobile Phone for___?	Very unlikely or unlikely *n (%)*	Uncertain *n (%)*	Very likely or likely *n (%)*	Comparisons (McNemar—Bowker test)
Answering and locating a ringing phone	124 (25)	157 (32)	203 (41)	AL-VC***; AL-LP***; AL-TB***
Voice call (handheld)	214 (44)	141 (29)	129 (26)	VC-LP***; VC-TB***
Looking at the phone continuously for more than 2 seconds	49 (10)	90 (18)	345 (71)	LP-TB^ns^
Texting/Browsing	45 (9)	89 (18)	350 (72)	

**Note:** AL-VC answering and locating a ringing phone- voice call comparison; AL-LP answering and locating a ringing phone- looking at the phone for continuously more than 2 seconds comparison; AL-TB answering and locating a ringing phone- texting/browsing comparison; VC-LP voice call- looking at the phone for continuously more than 2 seconds comparison; VC-TB voice call- texting/browsing comparison; LP-TB looking at the phone for continuously more than 2 seconds- texting/browsing comparison.

*** *p<* 0.001

^ns^ not significant

Frequency of responses for self-reported attitudes and task-management strategies for engaging in distracted driving by mobile phone task are shown in Table 4. Attitudes were statistically different between mobile phone conditions. Participants consistently reported that talking over the mobile phone was safer compared to texting or browsing (e.g. 12% of participants strongly agree or agree they would need a lot of convincing to believe talking is dangerous compared to the 4% who would need a lot of convincing about the danger of texting and browsing). With regard to the changes in driving behaviour to manage the secondary task of mobile phone usage, participants reported being very likely or likely to lower their driving speed (79%), increase their distance from the vehicle in front (70%), and scan the environment more often (44%) while texting or browsing. They were marginally more likely to report increasing their control over the steering wheel while talking (33%). With regard to changes in driving behaviour performed to avoid police enforcement, no differences were found between talking and texting/browsing in the proportion of participants who were very likely or likely to scan the environment for police (69–72%). Other behavioural changes, such as covering the phone all the time with your hand, were more likely to be reported while texting or browsing.

**Table 4:** Self-reported attitudes and task-management strategies for mobile phone distracted driving.

Items	Talking			Texting or browsing			Differences (McNemar—Bowker test)
**Attitudes towards mobile phone distracted driving**	***Strongly disagree or disagree n (%)***	***Neutral n (%)***	***Strongly agree or agree n (%)***	***Strongly disagree or disagree n (%)***	***Neutral n (%)***	***Strongly agree or agree n (%)***	
Easy for someone to tell if their driving has been affected	150 (31)	124 (26)	210 (43)	117 (24)	33 (7)	334 (69)	*P <* .001***
Need a lot of convincing to believe it is dangerous	347 (72)	81 (17)	56 (12)	456 (94)	8 (2)	20 (4)	*P <* .001***
Effects on driving ability are likely to be only very minor	268 (55)	104 (21)	112 (23)	431 (89)	21 (4)	32 (7)	*P <* .001***
Only people at risk are those who use a mobile while driving	427 (88)	30 (6)	27 (6)	439 (91)	17 (4)	28 (6)	*P* = .02*
Distraction effects will last even after the task is finished	126 (26)	142 (29)	216 (45)	98 (20)	129 (27)	257 (53)	*P <* .001***
Demanding driving conditions will prevent me from…	36 (7)	59 (12)	389 (80)	21 (4)	51 (11)	412 (85)	*P* = .001**
Presence of law enforcement and risk of a fine will prevent me from…	41 (8)	61 (13)	382 (79)	17 (4)	39 (8)	428 (88)	*P <* .001***
It is completely safe because I am generally extra careful	339 (70)	83 (17)	62 (13)	413 (85)	44 (9)	27 (6)	*P <* .001***
**Task-management strategies for mobile phone dual-tasking**	***Very unlikely and unlikely n (%)***	***Uncertain n (%)***	***Very likely and likely n (%)***	***Very unlikely and unlikely n (%)***	***Uncertain n (%)***	***Very likely and likely n (%)***	
Lower your driving speed	153 (31)	94 (19)	237 (48)	44 (9)	56 (11)	384 (79)	*P <* .001***
Increase your distance from the vehicle in front	133 (28)	96 (19)	255 (53)	57 (11)	84 (17)	343 (70)	*P <* .001***
Scan the environment more often	184 (38)	96 (19)	204 (42)	212 (43)	59 (12)	213 (44)	*P <* .001***
Increase the control over the steering wheel	202 (41)	120 (24)	162 (33)	237 (48)	88 (18)	159 (32)	*P <* .001***
Keep your mobile phone low (e.g. in lap or on passenger seat) for avoiding police	83 (17)	50 (10)	351 (72)	57 (11)	52 (10)	375 (77)	*P <* .005**
Scan the environment for police	82 (16)	66 (13)	336 (69)	71 (14)	64 (13)	349 (72)	*P <* .05^ns^
Cover the phone all the time with your hand	260 (53)	107 (22)	117 (24)	237 (48)	108 (22)	139 (28)	*P <* .001 ***

* *p<* 0.05

** *p<* 0.01

*** *p<* 0.001

^ns^ not significant

### Relationship of personal characteristics, attitudinal information, and task-management strategies with mobile phone usage while driving

To address Aim 2, separate logistic regression models were fitted for self-reported mobile phone handheld conversation (any sub-component) and text/browse (any sub-component) engagement on a typical day (yes or no). Associations between the mobile phone tasks and personal/attitudinal characteristics of participants were determined. Personal characteristics included: being 25 years old or less (yes/no), gender (male/female), time with a valid licence (years), hours driving (hours/day), having at least one traffic offence in the last three years (yes/no), having at least one crash in the last three years (yes/no), driving purpose (mostly for work/mostly personal/mixture), and type of vehicle they usually drive (SUV-utility car/small-medium car). Attitudinal information included: the eight attitude items towards mobile phone usage while driving (1–5 Likert scale), a risk perception item for each mobile phone task (1–5 Likert scale), and seven items describing task-management strategies by mobile phone task (1–5 Likert scale). Parsimonious logistic regressions were determined using maximum likelihood estimation. Neither handheld conversations nor texting/browsing models showed lack of fit at a *p <* 0.05. The regression model that resulted and the odds ratio estimates for self-reported engagement are shown in Table 5.

**Table 5:** Logistic regression analysis: Predicting handheld conversations and texting/browsing engagement.

Variables	Odds Ratio	95% CI	*β*	SE	*z*	Sig.
***Likelihood of engagement in handheld conversations (any sub-component) while driving on a typical day*** ^a^						
**Hours driving per day**	1.48	[1.19, 1.86]	0.39	0.11	3.5	<0.001
**Years with licence**	0.97	[0.95, 0.99]	-0.02	0.01	-2.48	0.013
**At least one traffic offence in the last three years (1 = yes)**	3.59	[2.3, 5.62]	1.27	0.22	5.61	<0.001
**At least one crash in the last three years (1 = yes)**	0.52	[0.32, 0.84]	-0.65	0.24	-2.64	0.008
**Attitudes:** *Distraction effects will last even after the task is finished*	0.81	[0.65, 0.99]	-0.21	0.1	-2.01	0.044
**Attitudes:** *Demanding driving conditions will prevent me from conversing*	0.67	[0.53, 0.84]	-0.39	0.11	-3.48	<0.001
**Perceived crash risk**	0.73	[0.6, 0.9]	-0.3	0.1	-2.97	0.003
**Task-management strategy:** *Scan the environment more often*	1.42	[1.13, 1.79]	0.35	0.11	3.03	0.002
**Task-management strategy:** *Keep your mobile phone low*	1.52	[1.15, 2.00]	0.41	0.14	2.99	0.003
**Task-management strategy:** *Cover the phone all the time with your hand*	0.65	[0.5, 0.84]	-0.42	0.13	-3.25	0.001
**Constant**	4.42	[1.04, 18.8]	1.48	0.73	2.02	0.044
***Likelihood of engagement in texting or browsing (any sub-component) while driving on a typical day*** ^b^						
**Hours driving per day**	1.23	[1.00, 1.50]	0.20	0.10	1.98	0.047
**Years with licence**	0.97	[0.94, 0.98]	-0.03	0.01	-3.58	<0.001
**At least one traffic offence in the last three years (1 = yes)**	1.66	[1.10, 2.50]	0.51	0.21	2.44	0.015
**Attitudes:** *It is completely safe because I am generally extra careful*	1.38	[1.10, 1.73]	0.33	0.11	2.86	0.004
**Task-management strategy:** *Scan the environment more often*	1.39	[1.12, 1.72]	0.33	0.11	3.07	0.002
**Task-management strategy:** *Scan the environment for police*	1.38	[1.05, 1.81]	0.32	0.14	2.32	0.02
**Constant**	0.12	[0.04, 0.28]	-2.13	0.45	-4.7	<0.001

^*a*^ Engagement in handheld conversation: Likelihood ratio Chi-Square = 110.5, *p*-value = *< 0*.*001; Bayesian Information Criterion (BIC) = 628*.*03; Akaike’s Information Criterion (AIC) = 582*.*02*

^*b*^ Engagement in texting/browsing: Likelihood ratio Chi-Square = 61.49, *p*-value = *< 0*.*001; Bayesian Information Criterion (BIC) = 652*.*73; Akaike’s Information Criterion (AIC) = 623*.*46*

#### Logistic regression summary for handheld conversations

Participants who drive for a large number of hours, reported at least one traffic offence in the last three years, reported being more likely to scan the environment more often, and reported being more likely to keep the phone low, have higher odds of reporting handheld mobile phone use on a typical day.

Specifically: for every additional hour driven per day, drivers had 1.48 times the odds of reporting engagement in handheld conversations (95% CI = 1.19–1.86); drivers who reported having at least one traffic offence in the last three years had 3.59 times the odds of self-reporting handheld conversations (95% CI = 2.30–5.62); drivers had 1.42 times the odds of reporting engagement in handheld conversations for every additional unit in the likelihood of scanning the environment more often (95% CI = 1.13–1.79); and 1.52 times the odds of reporting engagement in handheld conversations for every additional unit in the self-reported likelihood to keep the phone low (95% CI = 1.15–2.00).

Drivers who had their licence for a longer period, reported having a crash in the last three years, have a high perception of risk with regard to the task, believed that the effects of distraction are long-lasting even after the task is finished, consider that demanding conditions would activate self-regulation, and self-reported being likely to cover the phone all the time with their hand, were less likely to report engagement in handheld conversations.

Specifically, for every additional year of having a valid licence, the odds of self-reporting handheld conversations while driving decrease by 3% (95% CI = 0.95–0.99). Drivers who reported having at least one crash in the last three years had 0.52 times the odds of self-reporting handheld conversations (95% CI = 0.32–0.84). When the attitude that distraction effects will last even after the task is finished increased by one unit, the odds of self-reporting a crash are approximately 19% lower (95% CI = 0.65–0.99). When the belief that demanding driving conditions will prevent the driver from conversing increased by one unit, the odds of self-reporting a crash are approximately 33% lower (95% CI = 0.53–0.84). When drivers increased by one unit the perceived crash risk, the odds of self-reporting a crash are approximately 27% lower (95% CI = 0.60–0.90). Drivers had 0.65 times the odds of reporting engagement in handheld conversations for every additional unit in the likelihood of covering the phone with the hand (95% CI = 0.50–0.84)

#### Logistic regression summary for texting/browsing

Participants who drive for extended periods of time, reported at least one traffic offence in the last three years, declared high safe attitudes as a result of being “extra careful”, reported higher likelihood to scan the environment more often and scanning for police more often, have higher odds of self-reporting texting/browsing on a typical day.

Specifically, for every additional hour driven per day, drivers had 1.23 times the odds of reporting engagement in texting/browsing (95% CI = 1.00–1.50) and, if the driver reported having at least one traffic offence in the last three years, had 1.66 times the odds of self-reporting texting/browsing (95% CI = 1.10–1.73). When safe attitudes as a result of being “extra careful” increased by one unit, the odds of texting/browsing on a typical day were 38% higher (95% CI = 1.10–1.73). When self-reports of likelihood of scanning the environment more often increased by one unit, the odds of texting/browsing on a typical day were nearly 39% higher (95% CI = 1.12–1.72). When self-reported high likelihood of scanning the environment for police increased by one unit, the odds of texting/browsing on a typical day were nearly 39% higher (95% CI = 1.12–1.72).

Drivers who had held a licence for a longer time were less likely to report engagement in texting/browsing and conversations. For every additional year of having a valid licence, the odds of self-reporting handheld conversations while driving decrease by 3% (95% CI = 0.94–0.98).

## Discussion

Among a general driving population from Queensland (Australia), we surveyed differences in behaviour, attitudes and perceptions regarding mobile phone distracted driving. On a typical day, fewer drivers were likely to report talking on handheld mobile phone or texting/browsing than the other two surrogate measures of mobile phone distraction: looking at the screen for more than 2 seconds, or locating and answering a ringing phone. This is not surprising given that previous literature has confirmed that drivers are likely to partition mobile phone interactions (e.g. a study in the U.S. confirmed that most drivers receiving calls while driving just answer and keep driving [17]). Despite the plethora of interventions on mobile phone distracted driving, nearly one in every two respondents confirmed engaging in at least one of these tasks on a typical day. This situation is rather concerning, given that the tasks evaluated here at least double the odds of crash [5, 14]. Moreover, mobile phone engagement is likely to be higher because a wide range of mobile phone tasks, that could be performed by drivers, were not included in this study (e.g. take selfies or play games [18]).

In general, the most frequent interactions reported by drivers in Queensland involve heavy visual workload. Similar findings have been reported in the U.S. [19], Israel [20], and Australia (New South Wales) [21]. This is worrisome given that there is a large volume of published studies linking visual distraction with an increased crash risk [12]. A further examination confirms that, among the behaviours examined, looking for more than 2 seconds at a mobile phone while driving is the most common and frequent task while driving. A recent qualitative study by Gauld, Lewis [22] suggests that texting sub-tasks (e.g. initiating, monitoring/reading, and responding) have different underlying motivations. It can thus be suggested that focusing on dangerous human-machine interactions with the mobile phone rather than generic tasks, i.e. dangers of taking the eyes off the road or reducing the quality of visual information procurement, should be utilised to design more efficient enforcement strategies and communicate risks to the general public. Nonetheless, having a small frequency of drivers reporting engagement in texting/browsing can be partially attributed to social desirability bias given these tasks have been frequently the focus of road safety intervention and are unlawful activities in Queensland.

Levels of perceived risk were higher and comparable between looking at the phone for more than 2 seconds and texting/browsing. Mobile phone conversations were the tasks perceived as less risky. There are similarities between the levels of mobile phone conversations and texting/browsing risk perception expressed in this study and those described by Terry and Terry [19], Young and Lenné [23], Nelson, Atchley [24], and White, Eiser [25]. On the other hand, locating and reaching a ringing phone was perceived as having a risk around the mid-range between these tasks. An issue emerging from these results is that naturalistic observations of locating and reaching a ringing phone have revealed this to be one of the most risky activities that a driver could possibly engage in [5]. This mismatch in risk perception requires further research to understand its occurrence and implications for road safety.

Attitudinal and task-management self-reports were evaluated for talking and texting/browsing while driving. Drivers reported less positive attitudes for texting/browsing than mobile phone conversations. This is consistent with previous attitudinal research on mobile phone distracted driving [17]. Drivers in the sample reported being willing to stop using the phone to avoid police or when the complexity of the driving task was high. This suggests that drivers are engaging in tactical compensatory strategies that have been theorised in the driving behaviour literature for both workload management and police enforcement avoidance [26–28]. In addition, task-management strategies were more likely to be reported for texting/browsing. No differences were found in the item with regard to increasing the frequency of scanning the environment for police, suggesting that drivers are likely to have similar responses to police enforcement independently of the task.

Previous research in Australia confirmed that “reduce speed” is one of the frequent strategies used by drivers (78% of drivers; n = 158) to engage in other tasks [23]. A contribution of this research is confirmation that the task-management strategies might vary from task to task. The most frequent driving strategies for engaging in dual-tasking were increasing headway distance while talking (53%) and lowering driving speed while texting/browsing (79%). In reviewing the literature, naturalistic and simulator studies have consistently observed these behavioural responses [29–31]. Efforts to calibrate driver behavioural models and include human factors into traffic and transportation models are required to deal with these potential differences in drivers’ decision-making process.

Logistic regression models were fitted to determine predictors of mobile phone usage for talking (any sub-component) and texting/browsing (any sub-component) on a typical day. In both cases drivers who have held a valid driving licence for less time and spend more time driving per day were more likely to report engagement in the tasks. Previous research in the U.S. has shown that prevalence of secondary tasks with heavy visual demands increased over time among novice drivers but not among experienced drivers [32]. In this study, there is a perfect storm of distracted novice drivers who do not have the driving experience needed to engage in a secondary task. Naturalistic driving studies have showed that young adults aged 16–20 are at higher crash risk (OR: 3.53; overall cell use) than drivers aged 30–64 (OR: 2.11; overall cell use) [33]. This could be explained in terms of research which has indicated that less driving experience could be associated with a reduced amount of spare capacity needed to engage in inattentive driving [34],

Although years with a valid driving license was a significant predictor of texting/browsing and handheld talking while driving on a typical day, age alone did not predict mobile phone use (these variables were tested independently due to intercorrelation). Additionally, no gender differences were found in likelihood of reporting mobile phone engagement. These somewhat surprising results are consistent with previous research in other jurisdictions [35, 36]. Nonetheless, there are some unresolved questions of interest related to the role of individual differences in the distracted driving decision-making process. For instance, empirical evidence suggests that individual differences have a significant in situation-complexity assessment [37], risky behaviour of young novice drivers [38], and self-regulation practices [29]. These issues will be dealt with in another paper under preparation using the same data set, and are expected to explain the difference in crash risk between groups of drivers (e.g. young and mature drivers).

Having at least one traffic offence in the last three years was a predictor of actual self-reported usage of mobile phone while driving for texting/browsing and talking. Research elsewhere has reported similar results (see Márquez, Cantillo [39]). Recidivism might play a role and brings new opportunities for developing targeted interventions for this high-risk group.

As expected, attitudes were predictors of mobile phone engagement on a typical day for both models. Previous research has confirmed the role of attitudes and safety beliefs in the prediction of mobile phone distracted driving behaviour [28, 35]. It is important to highlight that attitudes related to the perceived capacity of self-regulation (i.e. to avoid mobile phone use in case of high driving demands and to adjust driving behaviour accordingly to the driving demands) seem to influence engagement on mobile phone distracted driving. These results imply that strategic (Would I use the mobile phone while driving?) and tactical (Under which circumstances would I use the mobile phone while driving?) mobile phone engagement decisions must be considered when investigating predictors of mobile phone distracted driving. Appropriate caution should be exercised in the interpretation of this finding because there is no guarantee that the drivers’ judgement of a driving situation is adequate to minimise crash risk [34, 40].

Perceived crash risk was a predictor of handheld conversation but not texting/browsing. Two explanations could be considered: first, although usually assumed otherwise (see Young, Rudin-Brown [8] on-road study showing a higher prevalence of handheld conversations than hands-free devices in Australia), it is likely that the majority of the drivers used a hands free interface which is not prohibited in Queensland and, therefore, considered safe by the public. Second, driver behaviour models have suggested that drivers have a threshold awareness risk [37]. As the complexity of texting/browsing compared to talking has been consistently perceived to be high, these tasks might have reached a threshold where the drivers might need to initiate a capability assessment.

The self-reported likelihood of engaging in task-management strategies was a predictor of mobile phone involvement on a typical day for both mobile phone tasks. Scanning the environment more often was a consistent predictor in the models, indicating that the decision to engage in mobile phone use is closely related to the perception of potential hazards. It is possible to hypothesise that drivers do not decide to engage in a secondary task based on future risks but actual road hazards. Unsurprisingly, none of the task-management strategies related to vehicle control (e.g. speed selection or steering wheel control) were predictors of engagement. Typically, modification of driving behaviour occurs once the driver is performing a secondary task, which suggests that the decision to use the mobile phone is scenario-based with no prearranged strategy for workload management. Using naturalistic data, Tivesten and Dozza [41] confirmed that drivers change their glance behaviour while distracted depending on where they are driving. Future research is needed to confirm this.

Scanning for police officers was also significant in the talking and texting/browsing models. This suggested that an enforcement oriented policy has not been able to minimise mobile phone usage in cars successfully and new system-wide countermeasures are needed. Additionally, the fact that drivers learned that covering the phone with their hand will help them avoid a police offence while driving must be incorporated in planning for road safety strategies. Nonetheless, an open question here is the interaction between enforcement avoidance and collision avoidance. Some of the strategies used by drivers, such as texting with the phone in their laps, might put them in a higher risk since this is not an optimal position for viewing the screens while driving (see Wittmann, Kiss [42] and Alconera, Garcia [43]).

## Limitations

The use of self-report measurements in this study is a clear limitation given their susceptibility to socially desirable responding or inaccurate memories. Although there is a chance that our findings might underestimate risky behaviours, the relatively high proportion of participants (50%) who reported high levels of engagement in the riskier behaviours suggests that participants responded honestly to the questions. The current sample also had a lower proportion of men than women which may limit its generalisability to the wider male population. However, the proportion seems typical to similar studies in Queensland [44]. Next, although this study assessed both psychological and physiological personal variables, there are other environmental variables that influence mobile phone usage while driving, such as heavy traffic and urbanization [29]. Finally, it is possible that the engagement of the allegedly risky behaviour studied in this research does not compromise safety. Human-factors theories, such as the minimum required attention (MiRA) theory [34], considers a driver should only be considered inattentive when the driving task is compromised, regardless of whether the driver is concurrently executing an additional task or not. Further discussion of this dispute is necessary to understand the impact of mobile phone distracted driving.

## Conclusions

Despite such limitations, the findings provide support for focusing on the development of injury prevention strategies as a result of mobile phone distracted driving. Drivers are engaging in risky tasks that have been consistently linked with an increased crash risk (Objective 1). A reconceptualization of the delivery of health messages is necessary and important to tackle all the different opportunities in which an undesirable interaction with mobile phones compromises safety. While typical behaviours such as texting and calling were low, the sub-tasks (with equal or more risk) were highly reported and, in the case of locating and answering a ringing phone, erroneously assessed as less risky. This phenomenon might reflect a lack of safety literacy among the drivers. Additionally, drivers reported more safety attitudes towards talking on a mobile phone and more frequent engagement in task-management strategies for texting/browsing.

Logistic regression models were fitted to understand the role of personal, attitudinal, perceptual, and behavioural information in mobile phone engagement while driving (Objective 2). Predictors of mobile phone engagement were intrinsically different between talking and texting/browsing. For both behaviours, novice drivers and frequent drivers were more likely to engage in distracted driving. Additionally, previous offences seem to predict mobile phone usage which suggests that this is a high-risk group to be considered in interventions. Generally speaking, drivers who intentionally use strategies to evade police detection were more likely to report engagement in distracted driving. This has important implications because enforcement of distracted driving is already difficult and expensive, and system-wide solutions are necessary to prevent the consequences of distracted driving. Nevertheless, the development of safe attitudes and improvement of risk-literacy could help in reducing the crash risk.

Based on the results from this study, other potential opportunities to formulate countermeasures include: (*i*) re-engineer mobile phone tasks to avoid sub-tasks that require visual demands (e.g. Windscreen Head-Up Displays (HUDs) project information onto the vehicle windscreen in line with the driver’s forward line of sight), (*ii*) restructure enforcement strategies to overcome behavioural adaptations such as keeping the mobile phone low and searching for police (e.g. technologies to support automated enforcement through the detection of radio frequencies), and (*iii*) educational campaigns could be designed to prevent visual interfaces while driving. Participants in this project showed at least two main misunderstanding: First, there is a mismatch between objective and subjective risk that needs to be corrected. The perceived crash risk reflects that the knowledge transmitted to drivers through campaigns or media have not been evidence-based. Second, counterintuitively, participants reported less engagement in texting/browsing than other tasks with similar or higher visual demands. Educational campaigns focusing on tasks such as texting and browsing perhaps have not been sufficient to deter engagement in mobile phone subtasks such as searching for a ringing phone or dialling a phone number. These considerations must be considered in future community programs and advertising campaigns.

In an effort to move toward a theoretical framework for guiding future research, these findings reflect and support the viability of using driver behavioural models to explain how drivers adapt their behaviour to changes in complexity. Particularly, the study of where drivers engage in mobile phone activities could benefit from utilisation of behavioural adaptation theory (See Young and Regan (27) and Oviedo-Trespalacios, Haque (26)).

## Supporting information

S1 Dataset Data used. (XLSX)

## References

[1] World Health Organization. Global status report on road safety 2015. Geneva: 2015 9241565063.

[2] Oviedo-Trespalacios O, Haque MM, King M, Washington S, editors. Influence of road traffic environment and mobile phone distraction on the speed selection behaviour of young drivers. Driver Distraction and Inattention Conference DDI 2015; 2015; Sydney.

[3] Haque MM, Oviedo-Trespalacios O, Debnath A, Washington S. Gap Acceptance Behavior of Mobile Phone—Distracted Drivers at Roundabouts. Transportation Research Record: Journal of the Transportation Research Board. 2016;2602:43–51. 10.3141/2602-06

[4] Pless C, Pless B. Mobile phones and driving. BMJ. 2014;348. 10.1136/bmj.g1193 24496306

[5] Dingus TA, Guo F, Lee S, Antin JF, Perez M, Buchanan-King M, et al Driver crash risk factors and prevalence evaluation using naturalistic driving data. Proceedings of the National Academy of Sciences. 2016;113(10):2636–41. 10.1073/pnas.1513271113 26903657 PMC4790996

[6] Farmer CM, Klauer SG, McClafferty JA, Guo F. Secondary behavior of drivers on cell phones. Traffic Injury Prevention. 2015;16(8):801–8. 10.1080/15389588.2015.1020422 25793432

[7] Wilkinson ML, Brown AL, Moussa I, Day RS. Prevalence and correlates of cell phone use among Texas drivers. Preventive Medicine Reports. 2015;2:149–51. 10.1016/j.pmedr.2015.02.010. 26844064 PMC4721465

[8] Young KL, Rudin-Brown CM, Lenné MG. Look Who's Talking! A Roadside Survey of Drivers’ Cell Phone Use. Traffic Injury Prevention. 2010;11(6):555–60. 10.1080/15389588.2010.499442 21128183

[9] Tucker S, Pek S, Morrish J, Ruf M. Prevalence of texting while driving and other risky driving behaviors among young people in Ontario, Canada: Evidence from 2012 and 2014. Accident Analysis & Prevention. 2015;84:144–52. 10.1016/j.aap.2015.07.011. 26344898

[10] Scott-Parker B, Oviedo-Trespalacios O. Young driver risky behaviour and predictors of crash risk in Australia, New Zealand and Colombia: Same but different? Accident Analysis & Prevention. 2017;99, Part A:30–8. 10.1016/j.aap.2016.11.001. 27865138

[11] Wundersitz LN. Phone Use While Driving: Results From an Observational Survey. Traffic Injury Prevention. 2014;15(6):537–41. 10.1080/15389588.2013.843075 24867565

[12] Oviedo-Trespalacios O, Haque MM, King M, Washington S. Understanding the impacts of mobile phone distraction on driving performance: A systematic review. Transportation Research Part C: Emerging Technologies. 2016;72:360–80.

[13] Wickens CD. Multiple resources and mental workload. Human Factors: The Journal of the Human Factors and Ergonomics Society. 2008;50(3):449–55. 10.1518/001872008X288394 18689052

[14] Simons-Morton BG, Guo F, Klauer SG, Ehsani JP, Pradhan AK. Keep your eyes on the road: Young driver crash risk increases according to duration of distraction. Journal of Adolescent Health. 2014;54(5):S61–S7. 24759443 10.1016/j.jadohealth.2013.11.021 PMC3999409

[15] Fitch G, Soccolich S, Guo F, McClafferty J, Fang Y, Olson R, et al. The impact of hand-held and hands-free cell phone use on driving performance and safety-critical event risk. Washington, DC: National Highway Traffic Safety Administration, 2013.

[16] Firth D. Bias reduction of maximum likelihood estimates. Biometrika. 1993;80(1):27–38.

[17] Tison J, Chaudhary N, Cosgrove L. National phone survey on distracted driving attitudes and behaviors. Washington, DC: National Highway Traffic Safety Administration, 2011.

[18] Postelnicu C-C, Machidon O-M, Girbacia F, Voinea G-D, Duguleana M, editors. Effects of playing mobile games while driving. International Conference on Distributed, Ambient, and Pervasive Interactions; 2016: Springer.

[19] Terry CP, Terry DL. Distracted Driving Among College Students: Perceived Risk Versus Reality. Current Psychology. 2016;35(1):115–20. 10.1007/s12144-015-9373-3

[20] Musicant O, Lotan T, Albert G. Do we really need to use our smartphones while driving? Accident Analysis & Prevention. 2015;85:13–21. 10.1016/j.aap.2015.08.023 26364139

[21] Waddell LP, Wiener KKK. What's driving illegal mobile phone use? Psychosocial influences on drivers' intentions to use hand-held mobile phones. Transportation Research Part F: Traffic Psychology and Behaviour. 2014;22:1–11. 10.1016/j.trf.2013.10.008

[22] Gauld CS, Lewis IM, White KM, Watson B. Key beliefs influencing young drivers’ engagement with social interactive technology on their smartphones: A qualitative study. Traffic Injury Prevention. 2016;17(2):128–33. 10.1080/15389588.2015.1047014 26528733

[23] Young KL, Lenné MG. Driver engagement in distracting activities and the strategies used to minimise risk. Safety Science. 2010;48(3):326–32. 10.1016/j.ssci.2009.10.008.

[24] Nelson E, Atchley P, Little TD. The effects of perception of risk and importance of answering and initiating a cellular phone call while driving. Accident Analysis & Prevention. 2009;41(3):438–44. 10.1016/j.aap.2009.01.006. 19393790

[25] White MP, Eiser JR, Harris PR. Risk perceptions of mobile phone use while driving. Risk Analysis. 2004;24(2):323–34. 10.1111/j.0272-4332.2004.00434.x 15078303

[26] Oviedo-Trespalacios O, Haque MM, King M, Washington S. Self-regulation of driving speed among distracted drivers: An application of driver behavioural adaptation theory. Traffic Injury Prevention. 2017;18:01–7. 10.1080/15389588.2017.1278628 28095026

[27] Young KL, Regan MA. Defining the relationship between behavioural adaptation and driver distraction In: Rudin-Brown C, Jamson S, editors. Behavioural Adaptation and Road Safety Theory, Evidence and Action. Boca Raton: CRC Press, Taylor and Francis Group 2013 p. 227–43.

[28] Walsh SP, White KM, Hyde MK, Watson B. Dialling and driving: Factors influencing intentions to use a mobile phone while driving. Accident Analysis & Prevention. 2008;40(6):1893–900. 19068291 10.1016/j.aap.2008.07.005

[29] Oviedo-Trespalacios O, Haque MM, King M, Washington S. Effects of Road Infrastructure and Traffic Complexity in Speed Adaptation Behaviour of Distracted Drivers. Accident Analysis & Prevention. 2017;101:67–77. 10.1016/j.aap.2017.01.018 28189943

[30] Saifuzzaman M, Zheng Z, Mazharul Haque M, Washington S. Revisiting the Task—Capability Interface model for incorporating human factors into car-following models. Transportation Research Part B: Methodological. 2015;82:1–19. 10.1016/j.trb.2015.09.011.

[31] Metz B, Schömig N, Krüger H-P. Attention during visual secondary tasks in driving: Adaptation to the demands of the driving task. Transportation Research Part F: Traffic Psychology and Behaviour. 2011;14(5):369–80.

[32] Klauer SG, Guo F, Simons-Morton BG, Ouimet MC, Lee SE, Dingus TA. Distracted driving and risk of road crashes among novice and experienced drivers. New England journal of medicine. 2014;370(1):54–9. 10.1056/NEJMsa1204142 24382065 PMC4183154

[33] Guo F, Klauer SG, Fang Y, Hankey JM, Antin JF, Perez MA, et al The effects of age on crash risk associated with driver distraction. International Journal of Epidemiology. 2017;46(1):258–65. 10.1093/ije/dyw234 28338711

[34] Kircher K, Ahlstrom C. Minimum required attention: a human-centered approach to driver inattention. Human factors. 2016;59(3):0018720816672756. 10.1177/0018720816672756 27738279

[35] Zhou R, Yu M, Wang X. Why Do Drivers Use Mobile Phones While Driving? The Contribution of Compensatory Beliefs. PloS one. 2016;11(8):e0160288 10.1371/journal.pone.0160288 27494524 PMC4975505

[36] Oviedo-Trespalacios O, Scott-Parker B. The sex disparity in risky driving: A survey of Colombian young drivers. Traffic Injury Prevention. 2017:00-. 10.1080/15389588.2017.1333606 28548584

[37] Lewis-Evans B, Rothengatter T. Task difficulty, risk, effort and comfort in a simulated driving task—Implications for Risk Allostasis Theory. Accident Analysis & Prevention. 2009;41(5):1053–63. 19664445 10.1016/j.aap.2009.06.011

[38] Scott-Parker B, Watson B, King M. Understanding the psychosocial factors influencing the risky behaviour of young drivers. Transportation Research Part F: Traffic Psychology and Behaviour. 2009;12(6):470–82. 10.1016/j.trf.2009.08.003.

[39] Márquez L, Cantillo V, Arellana J. Mobile phone use while driving: A hybrid modeling approach. Accident Analysis & Prevention. 2015;78:73–80. 10.1016/j.aap.2015.02.016. 25746167

[40] Huth V, Brusque C. Drivers' adaptation to mobile phone use: interaction strategies, consequences on driving behaviour and potential impact on road safety In: Stevens A, Brusque C, Krems J, editors. Driver Adaptation to Information and Assistance Systems London: IET; 2013 p. 173–96.

[41] Tivesten E, Dozza M. Driving context and visual-manual phone tasks influence glance behavior in naturalistic driving. Transportation Research Part F: Traffic Psychology and Behaviour. 2014;26, Part A(0):258–72. 10.1016/j.trf.2014.08.004.

[42] Wittmann M, Kiss M, Gugg P, Steffen A, Fink M, Pöppel E, et al Effects of display position of a visual in-vehicle task on simulated driving. Applied Ergonomics. 2006;37(2):187–99. 10.1016/j.apergo.2005.06.002. 16118009

[43] Alconera AM, Garcia L, Mercado JC, Portus AJ. A Study on the Positioning of a Mounted Mobile Phone to Reduce Distraction While Driving Among Young Adults Advances in Human Aspects of Transportation: Springer; 2017 p. 361–70.

[44] Lennon A, Oviedo-Trespalacios O, Matthews S. Pedestrian self-reported use of smart phones: positive attitudes and high exposure influence intentions to cross the road while distracted. Accident Analysis & Prevention. 2017;98:338–47. 27825043 10.1016/j.aap.2016.10.028

